# Lung Support Service: Implementation of a Nationwide Text Message Support Program for People with Chronic Respiratory Disease during the COVID-19 Pandemic

**DOI:** 10.3390/ijerph192417073

**Published:** 2022-12-19

**Authors:** Rebecca Raeside, Anna C. Singleton, Allyson Todd, Stephanie R. Partridge, Karice K. Hyun, Helen Kulas, Sally L. Wootton, Marita T. Dale, Jennifer A. Alison, Zoe McKeough, Renae J. McNamara, Lissa Spencer, Christine Jenkins, Julie Redfern

**Affiliations:** 1 Engagement and Co-Design Research Hub, Sydney School of Health Sciences, Faculty of Medicine and Health, University of Sydney, Sydney, NSW 2145, Australia; 2 Department of Cardiology, Concord Repatriation General Hospital, Sydney, NSW 2137, Australia; 3 NSW Agency for Clinical Innovation, Sydney, NSW 2065, Australia; 4 Chronic Disease Community Rehabilitation Service, Northern Sydney Local Health District, Sydney, NSW 2103, Australia; 5 Discipline of Physiotherapy, Sydney School of Health Sciences, Faculty of Medicine and Health, University of Sydney, Sydney, NSW 2006, Australia; 6 Allied Health Research and Education Unit, Sydney Local Health District, Sydney, NSW 2050, Australia; 7 Department of Physiotherapy, Prince of Wales Hospital, Sydney, NSW 2031, Australia; 8 Woolcock Institute of Medical Research, Sydney, NSW 2037, Australia; 9 Department of Physiotherapy, Sydney Local Health District, Sydney, NSW 2050, Australia; 10 Institute of Respiratory Medicine, Royal Prince Alfred Hospital, Sydney, NSW 2050, Australia; 11 The George Institute for Global Health, University of New South Wales, Sydney, NSW 2042, Australia

**Keywords:** digital health, text messaging, implementation science, public health, pulmonary rehabilitation, chronic respiratory disease, COVID-19

## Abstract

Background: COVID-19 pandemic lockdowns led to the closure of most in-person pulmonary rehabilitation programs in Australia. Text message programs are effective for delivering health support to aid the self-management of people with chronic diseases. This study aimed to evaluate the implementation of a six-month pre-post text message support program (Texting for Wellness: Lung Support Service), and the enablers and barriers to its adoption and implementation. Methods: This mixed-methods pre-post study used the Reach, Effectiveness, Adoption, Implementation and Maintenance (RE-AIM) framework to evaluate the Texting for Wellness: Lung Support Service, which is an automated six-month text message support program that included evidence-based lifestyle, disease-self management and COVID-19-related information. Reach was measured by the proportion of participant enrolments and demographic characteristics. Adoption enablers and barriers were measured using text message response data and a user feedback survey (five-point Likert scale questions and free-text responses). Implementation was evaluated to determine fidelity including text message delivery data, opt-outs, and intervention costs to promote and deliver the program. Results: In total, 707/1940 (36.4%) participants enrolled and provided e-consent, with a mean age (±standard deviation) of 67.9 (±9.2) years old (range: 23–87 years). Of participants who provided feedback, (326/707) most ‘agreed’ or ‘strongly agreed’ that the text messages were easy to understand (98.5%), helpful them to feel supported (92.3%) and helped them to manage their health (88.0%). Factors influencing engagement included a feeling of support and reducing loneliness, and its usefulness for health self-management. Messages were delivered as planned (93.7% successfully delivered) with minimal participant dropouts (92.2% retention rate) and low cost ($AUD24.48/participant for six months). A total of 2263 text message replies were received from 496 unique participants. There were no reported adverse events. Conclusion: Texting for Wellness: Lung Support Service was implemented quickly, had a broad reach, with high retention and acceptability among participants. The program was low cost and required minimal staff oversight, which may facilitate future implementation. Further research is needed to evaluate the efficacy of text messaging for the improvement of lung health outcomes and strategies for long-term pulmonary rehabilitation program maintenance.

## 1. Introduction

Chronic respiratory diseases, including chronic obstructive pulmonary disease (COPD), chronic asthma and bronchiectasis, are among the most common non-communicable diseases worldwide [1]. In 2017, 545 million people globally [2] and approximately 7.4 million people in Australia had a chronic respiratory disease [3]. Pulmonary rehabilitation is considered ‘standard care’ for people with chronic respiratory diseases [4], where participants are provided with in-person supervised exercise training, education on self-management of their condition and psychological support in outpatient hospitals or community settings. Participants who attend pulmonary rehabilitation have been shown to have improved exercise capacity, health-related quality of life and dyspnoea compared with those who do not attend [5]. However, in March 2020, the World Health Organization declared the novel Coronavirus Disease (COVID-19) pandemic [6], which caused the closure of in-person pulmonary rehabilitation programs across Australia. People with COPD were at significantly higher risk of poor clinical outcomes from COVID-19 including hospitalization, intensive care admission and mortality [7]. Many pulmonary rehabilitation programs rapidly transitioned to telehealth delivery and a recent systematic review found similar health benefits to in-person programs, but there was heterogeneity between programs [8]. Not only were people with chronic respiratory diseases no longer able to access their usual in-person rehabilitation and support programs, but they were also at higher risk of severe morbidity and mortality due to COVID-19 infections [9]. 

Globally, 91% of people own a mobile phone [10]. In Australia, mobile phone ownership continues to rise, with 19.9 million smartphone users in 2017 [11]. Mobile phones provide an opportunity to deliver support and promote the self-management of health through mobile applications and text messages. However, text messaging is the most accessible and widest reaching mobile technology due to the low-cost to send and receive them (often free), without the need for internet connections which may be unstable in regional and remote areas. Mobile phone services currently reach 99% of the Australian population [12], whereas only 91% are active internet users [13]. Previous research has demonstrated that text messaging is an effective way to deliver health support to people with chronic diseases [14,15], but there is limited evidence for the use of text messaging to deliver health support to people with chronic respiratory diseases. For example, a systematic review found that only 3/26 studies used digital technologies to support self-management of health for people with COPD [16]. However, research shows that pulmonary rehabilitation participants are willing to use technology as part of their health management, with 85% of attendees regularly using mobile phones [17]. Furthermore, evaluating the implementation of a community-based text message program to support people with chronic respiratory conditions and their health and wellbeing during the COVID-19 pandemic has not been previously investigated. Therefore, this study aimed to evaluate the implementation of a nationwide six-month pre-post text message support program (Texting for Wellness: Lung Support Service) to support the health and wellbeing of people with chronic respiratory diseases during the COVID-19 pandemic in Australia. It was hypothesized that the Texting for Wellness: Lung Support Service would have widespread uptake and be useful, acceptable and engaging to people with chronic respiratory disease.

## 2. Methods

### 2.1. Study Design

A mixed-methods (quantitative and qualitative) evaluation of a six-month pre-post text message support program for people with chronic respiratory disease during the COVID-19 pandemic was conducted using the enhanced Reach, Effectiveness, Adoption, Implementation and Maintenance (RE-AIM) framework [18]. The study was approved by the University of Sydney Human Research Ethics Committee (Approval No: 2020/181), and informed electronic consent (e-consent) was received from all participants. 

### 2.2. Participants and Recruitment

Participants were eligible if they were: (i) an adult (≥18 years), (ii) diagnosed with a chronic respiratory disease, (iii) had an Australian mobile phone number, and (iv) able to provide informed e-consent in English. Participants were recruited from 29 April 2020 to 1 February 2021 through paid and unpaid advertisements on social media (Facebook, Instagram and Twitter), paid radio advertisements, and through unpaid strategies including the Lung Foundation Australia (website, emails, newsletter and social media), and emails from lung disease organizations and rehabilitation programs. Potential participants were instructed to either click a secure weblink, text the word ‘Lung’ to the study mobile phone number, or scan a Quick Response (QR) code, which would lead them to the secure study website that contained the participant information sheet, e-consent form and privacy statements that detailed how participants’ personal information would be stored securely to maintain confidentiality and be used only for research purposes. After providing e-consent, participants completed demographic data (sex, age, postcode, smoking status). Next, participants registered their preferred name (‘nickname’) and mobile number into the automated text message delivery software, which initiated the first message in the program.

### 2.3. Texting for Wellness: Lung Support Service Intervention

Participants received a ‘welcome to the study’ message, which encouraged them to save the study mobile number in their phone. Participants then received four to five text messages per week (free-of-charge) for six months. The message delivery was automated, delivering messages at random times and on random days to minimize habituation [19]. The message content was adapted from the integrated text messaging program, which was co-designed with consumer representatives, health professionals, and researchers [20]. The original message content included recommendations on healthy lifestyle and behaviours, disease self-management, medication adherence and smoking cessation. Edits included encouraging COVID-19-safety regulations in Australia, such as abiding by social distancing (maintaining a 1.5 metre distance from other people) and self-quarantine regulations and how COVID-19 could impact people with respiratory diseases [9]. Approximately 30% of messages included links to websites with up-to-date, evidence-based information. All message content was positively toned, and semi-personalized using participants’ ‘preferred name’ or ‘nickname’ (see example text messages in Supplementary Table S1). 

The program was two-way (replies allowed), and participants were informed they would receive a response within 72 h. One message asked a direct question about how they heard about the program (options: 1. Health professional 2. Facebook 3. Instagram 4. Lung Foundation Australia 5. Other please specify). For safety, a member of the research team monitored replies and responded when appropriate. Participants could opt-out at any time by replying ‘STOP’ or requesting to unsubscribe and they were removed from the program within 72 h. The final message included a secure weblink to a user feedback survey regarding the acceptability and utility of the program (described in detail below). This text message was resent by the research team after one month if participants had not completed the survey. 

### 2.4. RE-AIM Framework

Reach and representativeness was evaluated using the percentage of people who enrolled in the study after visiting the study website (n enrolled/n visited study website × 100) and participant demographics, including age (years), sex (male, female), smoking status (current smoker yes/no) and postcode. Postcode was used to determine participants’ Index of Relative Socio-economic Advantage and Disadvantage (IRSAD) [21], which codes postcodes into quintiles from 1 (most-disadvantaged area) to 5 (least disadvantaged area) and remoteness using the Australian Statistical Geography Standard of Remoteness Areas [22], categorised as living in a Major City, Inner Regional Australia, Outer Regional Australia, Remote Australia and Very Remote Australia.

Efficacy for improving health outcomes was evaluated in a multi-centre randomized controlled trial [18], including exercise capacity, clinical measures, lifestyle outcomes, quality of life, mood and medication adherence. 

Adoption enablers and barriers were evaluated at the setting-level based on where participants were recruited, and the participant-level based on: (i) text message response data: the number, and content of text message replies, including text, emojis (a small digital image or icon used to express an idea or emotion) or ‘reactions’, which occur when a participant clicks a text message and clicks ‘like’, ‘love’, or ‘emphasise’ and; (ii) user feedback survey: 13 questions, including 11 5-point Likert scale questions (1 = strongly disagree to 5 = strongly agree) regarding the perceived usefulness of the program (ease-of-understanding, supportiveness), participants’ motivation to manage their health (e.g., eating healthier or being physically active), whether participants shared the program with family, friends or medical professionals, and participants’ preferences for program characteristics (number of messages per week, time of day messages received, length of the program and formality of the language within the messages). The survey also included two free-text questions regarding what participants liked most and least about the program, and suggestions for improvement. Staff-level adoption included evaluating the number of text message replies that required a staff response and estimated staff time.

Implementation and Maintenance was evaluated by the fidelity of the intervention including: (i) Text message delivery data (29 April 2020–8 August 2021): The number of text messages successfully delivered or unsuccessfully delivered (bounced); (ii) retention rate and percentage of opt-outs (n opt-outs/n baseline × 100); (iii) direct intervention costs, including delivering the text messages, monthly fees for the automated text message delivery software and advertising fees; and (iv) cost of staff time to monitor and respond to incoming text message, at an annual salary of AUD$78,760 ($AUD43.27/hour).

### 2.5. Analyses

Categorical data, including the text message delivery data and quantitative feedback survey data, were summarised by frequencies and percentages and continuous data were summarised as means and standard deviations. Text message responses and free-text feedback survey responses were independently coded into themes using the framework approach of two researchers (AT, AS), and any disagreements were reviewed by a third reviewer (RR) and discussed until an agreement was reached [23]. 

## 3. Results

Within 1.5 months of the Australian COVID-19 lockdown on 23 March 2020, ethics approvals and legal review were obtained to adapt the integrated text messaging program [18] into the Texting for Wellness: Lung Support Service and began nationwide implementation through automated text message delivery software.

### 3.1. Reach and Representativeness

**Figure 1:**
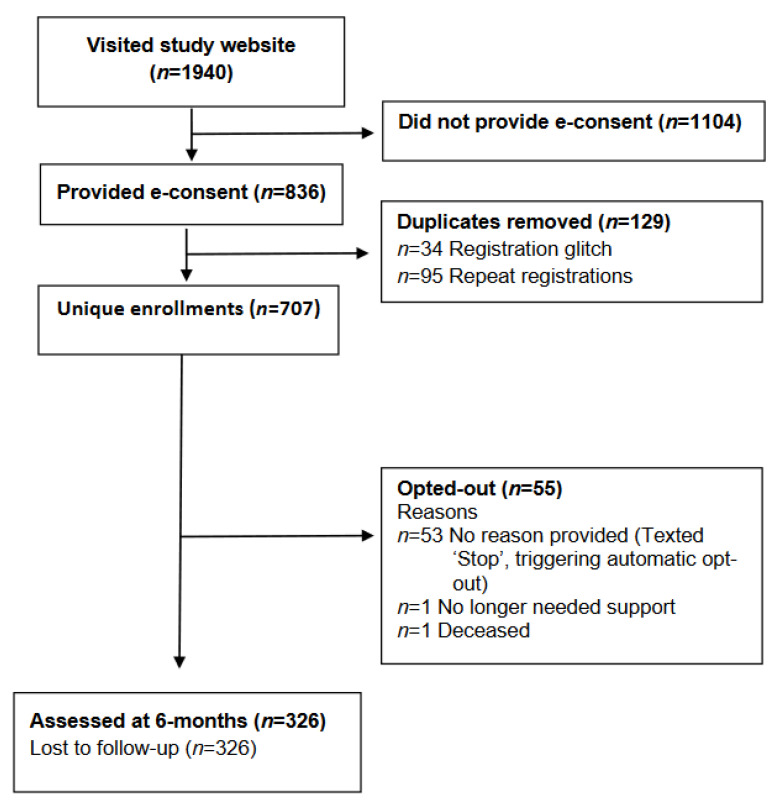
Study flow diagram.

A total of 1940 individuals visited the study registration website and 707 unique participants provided e-consent and completed the demographic data (707/1940; 36.4% enrolment rate; Figure 1). Participants had a mean (standard deviation) age of 67.9 ± 9.2 years (range: 23–87 years), most participants identified as female (501/707; 70.9%) and were not current smokers (589/707; 83.3%). All baseline participant characteristics are presented in Table 1. Participants enrolled from all states and territories in Australia; New South (259/706; 36.7%), Queensland (158/706; 22.4%), Victoria (138/706; 19.5%), Western Australia (66/706; 9.3%), South Australia (51/706; 7.2%), Australian Capital Territory (8/706; 1.1%), Tasmania (23/706; 3.2%) and Northern Territory (3/706; 0.4%). Based on participants’ postcodes, 17.1% (119/695) resided in Australia’s least disadvantaged areas (ISRAD quintile 5) and 21.9% (152/695) of participants lived in the most disadvantaged areas of Australia (quintile 1). Most participants lived in Major Cities (386/695; 55.5%) or Inner or Outer Regional (301/695; 43.3%), and few lived in Remote or Very Remote areas (8/695; 1.2%).

### 3.2. Enablers and Barriers to Adoption

Text message response data: 2263 text message replies from 496 unique participants were received. The median number of replies per participant was 2 (range 1–87) and 70% (496/707) of participants replied at least once. The most common replies were ‘thank you’ (603/2263; 26.6%), simple replies or emojis (507/2263; 22.4%), and the sharing of personal experiences (477/2263; 21.1%). Participants also shared how they had heard about the program (344/2263; 15.2%); nearly half of participants heard about the program through paid and unpaid social media advertising including Facebook (149/344; 43.3%) and Instagram (13/344; 3.8%). Other participants heard about the program through unpaid advertisements including Lung Foundation Australia (122/344; 35.5%), and from their healthcare professionals (17/344; 5.0%). Other replies included reactions to the message such as ‘like’ or ‘love’ (60/2263; 2.5%) or complimenting the program (20/2263; 0.9%), and some asked questions about the study, lung health or where to get additional support (82/2263; 3.6%) (see examples in Supplementary Box S1). The team sent 32 replies, including providing additional information about the study (9/32; 28.1%), directing participants to speak to a healthcare professional (10/32; 31.2%) or visit the Lung Foundation Australia website (5/32; 15.6%), confirming opt-out requests (3/32; 9.4%), or providing words of encouragement (4/32; 12.2%). Importantly, none of the replies were due to adverse events from the text messages. 

User feedback survey: 326/707 (46.1%) participants completed the user feedback survey (Figure 1), and quantitative responses are summarized in Table 2. Nearly all participants ‘agreed’ or ‘strongly agreed’ that the text messages were easy to understand, helped them feel supported and were useful, and most agreed the program helped them manage their health. Most (72.4%) participants felt the program length was ‘just right’, but 24.2% felt that six months was too short and wanted the option to continue. Some participants shared their messages with family (47.2%) or friends (29.1%). However, only a quarter (25.2%) shared with a medical professional (GP or specialist), and 32.2% did not share their messages with anyone.

#### 3.2.1. Engagement Enablers and Barriers

The thematic analysis of free-text survey responses mirrored the quantitative data, revealing two main factors influencing engagement: (1) experiencing healthcare support, and (2) usefulness of the program for managing health (Box 1). Sub-themes included feeling supported, mental health benefits of feeling less alone, and being motivated to improve health behaviours. Most participants felt the messages were informative and easy to understand, but some felt that the information was too general and did not improve their knowledge.

Box 1 **Factors influencing participants’ engagement with the program and suggestions for improvement.**

**THEME 1. EXPERIENCING HEALTHCARE SUPPORT**

**Feeling supported**
*Just letting me know you were always there to support me*—Female, age 41, Major City*It feels like someone is out there who truly cares and can depend on*—Male, age 64, Major City*It had the knack of providing the right support at exactly the right time*—Female, age 64, Inner Regional Australia
**Mental health benefits of feeling less alone**
*Although I’m aware the messages were generated electronically, it made me feel less alone. The support provided has been wonderful and I’m very grateful for being involved*—Female, age 64, Major City*I felt so much less alone by receiving these tips that though generic felt written in a very caring way specifically for me. Great for my mental well being too*—Female, age 70, Major City*I felt alone with my condition before I subscribed to the messages*—Female, age 64, Inner Regional Australia
**THEME 2. USEFUL FOR MANAGING MY HEALTH**

**Informative**
*Very informative and I learnt some new things I was unaware of*—Female, age 69, Inner Regional Australia*The messages gave me valid information, but not overloaded it*—Female, age 70, Very Remote Australia*Learnt some coping tips didn’t know. Main one, when breathless bend forward with arms resting in front of you*—Male, age 70, Major City
**Easy to understand**
*They were easy to understand and gave easy to follow instructions when needed*—Female, age 65, Major City*Even people with basic reading skills can follow these*—Female, age 65, Major City
**Gentle health reminders that motivated behaviour change**
*It was daily reminders of good things to do for myself that would help my quality of life. I thought they were great prompts*—Female, age 68, Inner Regional Australia*It reminded me of the very small things I can do to make a difference to my own lung health and wellbeing. Encouraged me to challenge myself and not get disheartened*—Female, age 70, Major City*They form a reassurance that exercise, diet, other simple strategies like breathing and recovery are in my control, and can benefit my condition*—Male, age 66, Major City
**SUGGESTIONS FOR PROGRAM IMPROVEMENT:**

**CONTROL OVER PROGRAM FEATURES**

**Program length**
*The only negative thing is that it has finished and I will no longer receive my comforting, helpful messages any longer*—Female, age 62, Major City*Loved the program, could only be improved by extending the program*—Female, age 66, Major City*The program should be ongoing: it is good for staying motivated*—Male, age 68, Outer Regional Australia
**Message frequency**
*I received daily messages… on the odd busy day I did ignore them till after work but always looked at them in the evening…so maybe an option of how many messages a week would be my only note*—Female, age 54, Inner Regional Australia*Too many messages. Sometimes up to 4 consecutive days. Preferable to have less and be able to focus on one thing more slowly*—Female, age 77, Major City
**More detailed information**
*I would like to see some advice about condition specific actions. Keeping it general weakens the type of help that this can provide*—Male, age 66, Inner Regional Australia*Could be more new information instead of basic information which people with COPD already know. It was useful as memory jogger*—Male, age 74, Major City*Some messages needed a little more detail*—Female, age 71, Inner Regional Australia

#### 3.2.2. Suggestions for Improvement

Based on the free-text survey feedback, some participants expressed a potential benefit of having control over program features, including the length of the program and message frequency, to suit their schedules and support needs. Some participants also wanted more details within the messages or specific practical advice regarding their lung health (Box 1).

### 3.3. Implementation and Maintenance: Program Fidelity and Costs

Fidelity: 83,573 text messages were sent from 29 April 2020 to 8 August 2021; 78,301 (93.7%) were delivered successfully, 5203 (6.2%) bounced, and 69 (<0.001%) were pending (neither delivered nor bounced). The retention rate for the program was 92.2% (652/707). Fifty-five (7.8%) participants opted out; 53 participants opted out by replying ‘STOP’ and two sent a text message reply requesting to opt out. Reasons for opt outs are summarised in Figure 1. 

Cost: The direct intervention costs of delivering Texting for Wellness: Lung Support Service to 707 participants was AUD$8269.84, including text message costs (AUD $6685.84) and automated message delivery software (AUD$1584). Advertising costs were AUD$5416.15, including social media (AUD$2916.15) and radio advertising (AUD$2500). Estimated costs for staff to monitor incoming messages and send minimal replies (approx. 75-min per week for 67 weeks; 83.75 h of work) was AUD$3623.86. Together, the total cost of promoting, delivering, and staff monitoring the program was AUD$17,309.85 or $24.48 per participant for six months.

## 4. Discussion

The period of the COVID-19 pandemic and its associated lockdowns has been a challenging time, especially for people living with chronic respiratory diseases whose in-person pulmonary rehabilitation programs were cancelled across Australia. The integrated text messaging [18] program was rapidly adapted to the Texting for Wellness: Lung Support Service to provide COVID-19-related health support and advice to people living with chronic respiratory diseases during this challenging time. To our knowledge, this is the first study of its kind among people with chronic respiratory diseases. This study used community-based recruitment strategies including social media advertising, emails and the Lung Foundation Australia to recruit 707 diverse individuals with a chronic respiratory disease from across Australia within nine months. The program was delivered as planned, and participants were engaged and found the program to be useful, helpful in providing support, and motivating for behaviour change, including physical activity and healthy eating. 

The use of digital technologies to deliver self-management programs for people with chronic respiratory conditions is an emerging area of research. Texting for Wellness: Lung Support Service was delivered solely by text messaging, as planned, with a high retention rate (652/707; 92.2%), minimal cost (AUD$24.48/participant for six months) and had a broad reach, with participants signing up from within a diverse age range (23–87 years old) and socioeconomic backgrounds and from all states and territories across Australia, including both urban and rural communities. This is representative of those who have chronic respiratory diseases in Australia [3]. Self-management programs delivered via text message have the potential to reach large numbers of the population at a low cost, [24] and are especially relevant in the context of the COVID-19 pandemic and associated lockdowns, as well as to people living in poorly resourced settings and remote areas. For example, the Text4Hope program was launched in Canada in 2020 to support people’s mental health during COVID-19 and found similar results, reaching 32,895 individuals in one week [25]. This study found community-based resource-light recruitment strategies were feasible, with 82.6% (284/344) hearing about the program through social media (Facebook, Instagram) and a national not-for-profit organization (Lung Foundation Australia) through their website, newsletters, social media and direct email to members. This supports previous research which has suggested that social media is an effective recruitment method for hard-to-reach populations [26]. This was especially true for this population, who were strongly advised to stay at home during the COVID-19 lockdowns. 

This study found that Texting for Wellness: Lung Support Service was feasible for overcoming common barriers to the implementation of health self-management support programs for people with chronic respiratory disease, including health literacy levels, insufficient healthcare practitioner time, resources or skills, [27] and psychological difficulties [28]. Of those who responded to the feedback survey, 98.5% of participants reported that the text messages were easy to understand, and 94.5% of participants said that the language was ‘just right’. Delivering health information that is easy to understand is an important resource for people to self-manage their conditions [29]. In addition, the day-to-day management of this program required little staff time (~75 min/week). The COVID-19 pandemic increased the global prevalence of depression and anxiety [30], the long-term impacts of which are still unknown. Although data on existing mental health diagnoses were not collected as a part of this study, the quantitative and qualitative feedback revealed that the program helped participants feel supported and less alone. The Text4Hope program found similar results with reductions in anxiety and stress levels after six weeks of daily messages during the COVID-19 pandemic [25]. Therefore, Texting for Wellness: Lung Support Service holds potential as a program to assist people to self-manage their health conditions and maintain good quality of life [16,31], overcoming common implementation barriers. 

This study is not without its limitations. Firstly, as this program was offered as a service, completion of the feedback survey was optional. The high retention rate (652/707; 92.2%) suggests participants liked the program, yet the user feedback survey had a 46% response rate and therefore the feedback may not be representative of all participants. A recent systematic review has demonstrated that online surveys have a response rate of around 46% and therefore this study is consistent with prior research [32]. Furthermore, though participants reported that the program provided motivation for physical activity and healthy eating, effectiveness in improving these outcomes was not explicitly measured. However, text message interventions have been found to be effective for the improvement of physical activity and healthy eating in other populations [15,33,34], and the results of the integrated text messaging program randomized controlled trial will provide evidence for effectiveness for people with chronic respiratory disease. Finally, although this program had strengths in reaching a diverse population and had many enablers of adoption (e.g., low cost and scalability), the long-term maintenance of the program still requires evaluation. To fully understand the impact of Texting for Wellness: Lung Support Service and continue its implementation within a community or hospital setting, ongoing funding will be required, along with further research to identify whether the program is effective in improving health outcomes. 

## 5. Conclusions

Texting for Wellness: Lung Support Service was rapidly implemented and had a broad reach, with high retention and acceptability among people with chronic respiratory conditions. Factors influencing engagement with the program included experiencing continued healthcare and its usefulness in health self-management. The program was low cost, factoring in both program delivery and staff time, which may assist with future implementation. Further research is needed to understand whether text message support improves health outcomes for people with chronic respiratory conditions and strategies for long-term program maintenance. 

## Figures and Tables

**Table 1:** Baseline participant characteristics.

	n/N (%)
Characteristic	Total N = 707
**Demographics**	
Age (years), mean (SD); range (min-max)	67.8 (9.2); 23–87
Gender	
Male	204/707 (28.9)
Female	501/707 (70.9)
**Current Smoker**	
Yes	118/707 (16.7)
No	589/707 (83.3)
**Index of Relative Socio-economic Advantage and Disadvantage [IRSAD], categorized from postcode**	
Quintile 1 (most disadvantaged)	152/695 (21.9)
Quintile 2	166/695 (23.9)
Quintile 3	155/695 (22.3)
Quintile 4	103/695 (14.8)
Quintile 5 (least disadvantaged)	119/695 (17.1)
**Australian Standard Geographical Classification Remoteness Area**	
Major City	386/695 (55.5)
Inner Regional Australia	222/695 (31.9)
Outer Regional Australia	79/695 (11.4)
Remote Australia	6/695 (0.9)
Very Remote Australia	2/695 (0.3)

**Table 2:** Perceived acceptability and usefulness of the Texting for Wellness: Lung Support Service.

Characteristic	No./Total (%) ^a^
**Usefulness ^b^**	
Found messages useful	303/326 (92.9)
Majority of messages were easy to understand	321/326 (98.5)
Program helped participant feel supported	301/326 (92.3)
**Motivation and health management ^b^**	
Messages helped me manage my health	287/326 (88.0)
Messages motivated me to be physically active	267/326 (81.9)
Messages motivated to eat healthier	239/324 (73.8)
**Message sharing**	
Friend	95/326 (29.1)
Family member	154/326 (47.2)
GP/doctor	58/326 (17.8)
Specialist	24/326 (7.4)
Nobody	105/326 (32.2)
**Program characteristics**	
Language of text messages ‘just right’ ^c^	308/326 (94.5)
Number of messages per week ‘just right’ ^d^	300/326 (92.0)
6-month program length was ‘just right’ ^e^	236/326 (72.4)
6-month program length was ‘too short’ or ‘much too short’ ^e^	79/326 (24.2)
Time of day receiving messages was appropriate ^b^	272/323 (84.2)

^a^ Response rate was 326/707 (46.1%) unique participants. ^b^ Response options were ‘strongly disagree, disagree, neutral, agree, strongly agree’. The proportion of participants who ‘strongly agree’ and ‘agree’ is reported. ^c^ Response options: ‘too casual, casual, just right, formal, too formal’. ^d^ Response options: ‘much too many, too many, just right, too few, much too few’. ^e^ Response options: ‘much too long, too long, just right, too short, much too short’.

## Data Availability

The data are not publicly available due to privacy to maintain confidentiality of participants who took part in this study.

## Supplementary Materials

The following supporting information can be downloaded at: https://www.mdpi.com/article/10.3390/ijerph192417073/s1, Table S1: Example text messages from Texting for Wellness: Lung Support Service; Box S1: Text message reply themes.
