# Supplementary material for: Lung Support Service: Implementation of a Nationwide Text Message Support Program for People with Chronic Respiratory Disease during the COVID-19 Pandemic
Source: Int J Environ Res Public Health. 2022 Dec 19;19(24):17073. doi: 10.3390/ijerph192417073 (PMC9779082; doi:10.3390/ijerph192417073)
Supplement: Supplementary file 1 [file ijerph-19-17073-s001.zip › Supplementary Box S1. Text responses_141222.pdf]

Supplementary Box S1. Text message reply themes.

**THEME 1: THANK YOU**
Thank you, I needed to hear that right now! – Female, age 67, Outer regional Australia
Thank you so much for the advice you share with me I don't feel so alone very grateful – Male, age 67, Outer regional Australia
Great link thank you. – Male, age 70, Major City
**THEME 2: SIMPLE REPLIES AND EMOJIS**
Okay – Male, age 71, Major city
👍 – Male, age 64, Inner regional Australia
Yes – Female, age 69, Inner Regional Australia
**THEME 3: SHARING PERSONAL EXPERIENCES**
**Following advice from the message**
Yes, I will do that thanks – Male, age 70, Major City
Thankyou this really worked for me. I'm not gasping for air when its hot and humid and hard to breathe 😉 – Female, age 68, Inner Regional Australia
Yes I have fan going on to my face it's a great help to get my breath – Female, age 72, Very remote Australia
**Exercise**
Today I have walked 13,000 steps (39 floors) and feeling good! 👫👫– Female, age 72, Major City
I pulled my bicycle out of storage!!! – Female, age 74, Outer Regional Australia
Just started a 8 week course exercise – Male, age 79, Major City
**Healthy Eating**
I love my veggies and fruit always have since a child 😉 – Female, age 59, Major City
I am on a fodmap diet due to IBS since my gall bladder was taken out so I am losing weight – Female, age 75, Inner Regional Australia
**Smoking**
I ain't a passive smoker and I haven't had a smoke for three days now – Female, age 68, Remote Australia
I am 77yrs old and have never had a cigarette in my mouth and I have COPD all through other people's smoking – Female, age 77, Major City
My homes in past 19years have been smoke free. Since i gave up smoking. – Female, age 67, Major City
**Personal challenges**
I haven't been well lately my RA and OA have been acting up again, weak and very tired every day I get tired very easily. I just thought I would write to you. Thank you for your help Very SOB on walking or anything else 👋 – Female, age 78, Major City
I'm too tired for any exercise at the moment – Male, age 79, Major City
I live in a small town, Doctors come and go every few months, therefore less interest. – Female, age 74, Outer Regional Australia
This is so sad for me. You are the only support I have. – Female, age 67, Inner Regional Australia
**COVID-19-related healthcare issues**
I have something to say at this point of time.. "because of Covid19, US the people who have Copd.. we HAVE not been able to attend our regular meetings with the regular team of nurses and physics, which I have found it VERY HARD..the GPs are finding it

very hard of how to tend MY issues.... – Female, age 76, Major City
**Enjoyed the program**
It's good to have someone to listen and tell my problems too I don't bother telling my family as I think they get sick of me – Female, age 78, Major City
Your messages have really helped me cope with the worst period of my life. – Female, age 76, Inner Regional Australia
**THEME 4: ASKING QUESTIONS**
**Study-related**
Can you text my mum messages like you send me as she has copd also – Female, age 41, Major City
Where is the feedback survey? (F, age 69, Inner Regional Australia)
Hi. I haven't had one of your super helpful texts for 2 days, I always look forward to them?? – Female, age 64, Inner Regional Australia
Is there a human on the other end of these messages? – Female, age 66, Major City
Why is it the last message - I have picked up some good tips – Female, age 71, Major City
**Exercise**
Did a big walk yesterday around Woolies does that count? – Female, age 81, Inner Regional Australia
Sweeping the paths in the garden does that count plus have been upstairs three times . – Female, age 75, Major City
**COPD management**
What are the breathing techniques please? – Female, age 68, Major City
Please explain pursed lip breathing again please. – Female, age 70, Outer Regional Australia
Ok. Was wondering with copd is it normal to feel really tired all the time. Have no energy at all. – Female, age 41, Major City
I want to know if it is ok to have the Covid vaccine given I have COPD, asthma and RA which is an autoimmune disease. I would appreciate any advice – Female, 75, Inner Regional Australia
**Where to find additional information**
Where do I find out about breathing techniques? – Female, age 64, Major City
Who am I supposed to ask? – Female, age 56, Inner Regional Australia
They say there is a new breakthrough, for asthma, when do we find out when it is available, do you know? – Female, age 69, Inner Regional Australia
