# Supplementary material for: Lung Support Service: Implementation of a Nationwide Text Message Support Program for People with Chronic Respiratory Disease during the COVID-19 Pandemic
Source: Int J Environ Res Public Health. 2022 Dec 19;19(24):17073. doi: 10.3390/ijerph192417073 (PMC9779082; doi:10.3390/ijerph192417073)
Supplement: Supplementary file 1 [file ijerph-19-17073-s001.zip › Supplementary Table S1. Example text messages_141222.pdf]

Supplementary Table S1. Example text messages from Texting for Wellness: Lung Support Service.

Message Topic	Example Text Message
Recommendations on healthy lifestyle and behaviours	Hi [pref_name][a], remember it is important to keep active - try a walk outside if possible but if not try walking around the garden or within your home
Disease self-management	When you are feeling breathless, follow the 3 Ps, Pause, Position, and Pursed lip breathing (stop, lean forward and breath slowly)
Medication adherence	Are you sure you're getting maximum benefit from your inhalers? For a demo on how to use your inhaler go to www.lungfoundation.com.au
COVID-19 advice	If you have to go out for essential items or to visit your doctor - keep a safe distance from others i.e. at least 1.5 metres apart

[a] [pref_name]: Participants preferred name entered at program enrolment.
